# Esophageal Rupture Presenting with ST Segment Elevation and Junctional Rhythm Mimicking Acute Myocardial Infarction

**DOI:** 10.1155/2021/8843477

**Published:** 2021-11-18

**Authors:** Wytch Rigger, Raymond Mai, P. Tim Maddux, Stuart Cavalieri, Joe Calkins

**Affiliations:** ^1^Department of Medicine, Medical College of Georgia at Augusta University, Augusta, GA, USA; ^2^Division of Cardiology, Medical College of Georgia at Augusta University, Augusta, GA, USA; ^3^Charlie Norwood VA Medical Center, Augusta, GA, USA

## Abstract

Esophageal rupture is a rare but potentially fatal cause of chest pain. The presentation is variable and can mimic other conditions such as aortic dissection, pulmonary embolism, and myocardial infarction (MI). A 71-year-old male with a history of coronary artery disease presented to the ED with complaints of acute chest pain and respiratory distress. Over the next 48 hours, the patient developed dynamic ST segment changes on surface electrocardiogram mimicking an inferolateral ST segment elevation MI accompanied by a junctional rhythm. Curiously, his cardiac enzymes remained negative during this time, but his clinical status continued to deteriorate. A subsequent CT scan demonstrated a lower esophageal rupture, and the patient underwent successful endoscopic stenting. While rare, prompt recognition of esophageal rupture is imperative to improving morbidity and mortality. While esophageal rupture has been noted to cause ST segment elevation before, this appears to be the first case associated with a junctional rhythm.

## 1. Introduction

Acute chest pain is a common patient presentation that carries a broad differential diagnosis.

Some of the more ominous diagnoses include myocardial infarction (MI), pulmonary embolism (PE), pancreatitis, peptic ulcer perforation, esophageal perforation, aortic aneurysm dissection, spontaneous pneumothorax, and pneumonia. Delineating between the aforementioned depends on the history, examination, laboratory, electrocardiogram (ECG), and imaging findings. Misdiagnosis can delay appropriate treatment, increase patient morbidity, and in some cases expedite death.

Boerhaave's Syndrome or spontaneous rupture of the esophagus is a rare disease. Nevertheless, it is a very dangerous and sometimes fatal as a patient's condition can rapidly deteriorate if a quick diagnosis is not made. Classically, Boerhaave's Syndrome is characterized by vomiting or retching followed by severe chest and/or abdominal pain, subcutaneous emphysema, and shortness of breath [1]. However, not all cases of spontaneous esophageal rupture are associated with vomiting [2]. Additional findings will include diminished breath sounds, abnormal imaging studies, and occasional nonspecific ECG abnormalities.

ST segment myocardial infarction (STEMI) is a clinical syndrome defined by symptoms of myocardial ischemia, together with ECG ST-segment changes, predominantly elevation, indicative of occlusion of a coronary artery. The diagnosis of MI is confirmed by evidence of myocardial necrosis based on biomarkers, ECG, or pathological examination. Like Boerhaave's Syndrome, STEMI is a medical emergency. While survival in STEMI continues to improve, the diagnosis continues to carry significant morbidity and mortality [3].

## 2. Case Report

**Figure 1:**
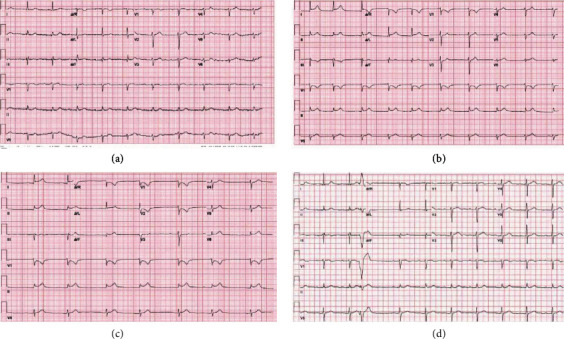
(a) Baseline ECG notable for 1^st^ degree AV block and poor R wave progression that was comparable to the patient's prior ECGs. (b) ECG on hospital day 2 showing the interval development of a junctional rhythm along with minor ST segment elevation in leads I, II, and aVL. (c) ECG 36 hours after admission showing persistence of the junctional rhythm along with ST segment elevation in leads I, II, III, aVL, aVF, and V6 along with ST segment depression in leads V1 and V2. This ECG prompted a code STEMI to be activated. (d) ECG after recovery showing a return to normal sinus rhythm with persistence of the 1^st^ degree AV block and a return of the ST segments to their original baseline.

A 71-year-old male with known multivessel coronary artery disease (CAD), paroxysmal atrial fibrillation, end-stage renal disease (ESRD) status postkidney transplant, hypertension, hyperlipidemia, and chronic GERD was admitted to the intensive care unit with severe chest pain and respiratory distress. The patient's symptoms were sudden in onset. The chest pain had a pleuritic component, and symptoms were exacerbated by straightening the lower limbs. Additional symptoms included nausea, vomiting, chills, and generalized weakness. Initial troponin was negative. Initial ECG at time of admission showed normal sinus rhythm, first-degree AV block, and poor R wave progression (Figure 1).

On hospital day two, cardiology was consulted to evaluate the patient given the aforementioned presentation and a change in the patient's ECG. The patient now had a junctional rhythm and nonspecific ST segment elevation in leads I and II (Figure 1). After evaluation, the patient's symptoms were felt to be inconsistent with typical angina. The patient's condition and symptomatology worsened, leaving the primary ICU team concerned for aortic dissection as the patient noted increasing pain radiating through his chest to his back. Given the patient's history of renal transplant and acute kidney injury during this admission, a transesophageal echocardiogram (TEE) was performed rather than a computed tomography (CT) scan in order to avoid contrast exposure. The patient was intubated for the study in the setting of poorly controlled pain and evolving respiratory distress. The TEE was negative for aortic dissection.

**Figure 2:**
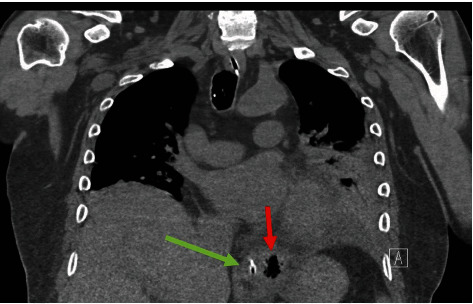
Noncontrasted coronal CT images showing a pocket of air in the mediastinum left lateral to the esophagus (red arrow) consistent with esophageal perforation. Green arrow identifies an NG tube within the esophageal lumen.

That evening, cardiology was called back to the patient's bedside to evaluate the patient for a STEMI. ECG at the time showed ST segment elevation in leads I, II, III, aVL, aVF, and V6 and ST segment depression in V1 and V2 (Figure 1). Coving of the ST segments was absent and cardiac enzymes remained negative. After multidisciplinary discussion, a noncontrasted CT scan was performed which revealed a distal esophageal rupture (Figure 2) complicating management of this critically ill patient who was now in shock. Further investigation and discussion uncovered a recent patient history of severe retching and vomiting prior to presentation at the emergency department. The patient's presenting symptoms and clinical findings were consistent with Boerhaave's Syndrome.

In the setting of the patient's abnormal ECG, a diagnostic coronary and bypass graft angiography was performed on hospital day 3 to rule out acute coronary syndrome. The study revealed a patent left internal mammary artery (LIMA) to left anterior descending artery (LAD) graft, patent right coronary artery (RCA), and patent circumflex coronary artery (LCx) system.

**Figure 3:**
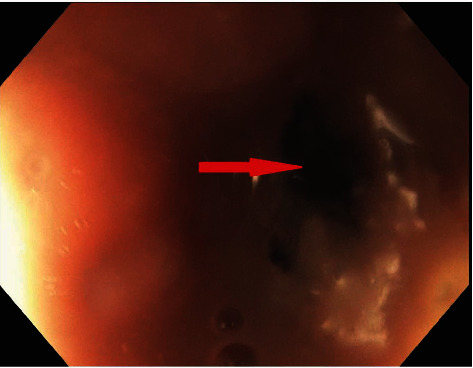
Endoscopic view of the distal esophageal perforation was identified at 31 cm (red arrow).

**Figure 4:**
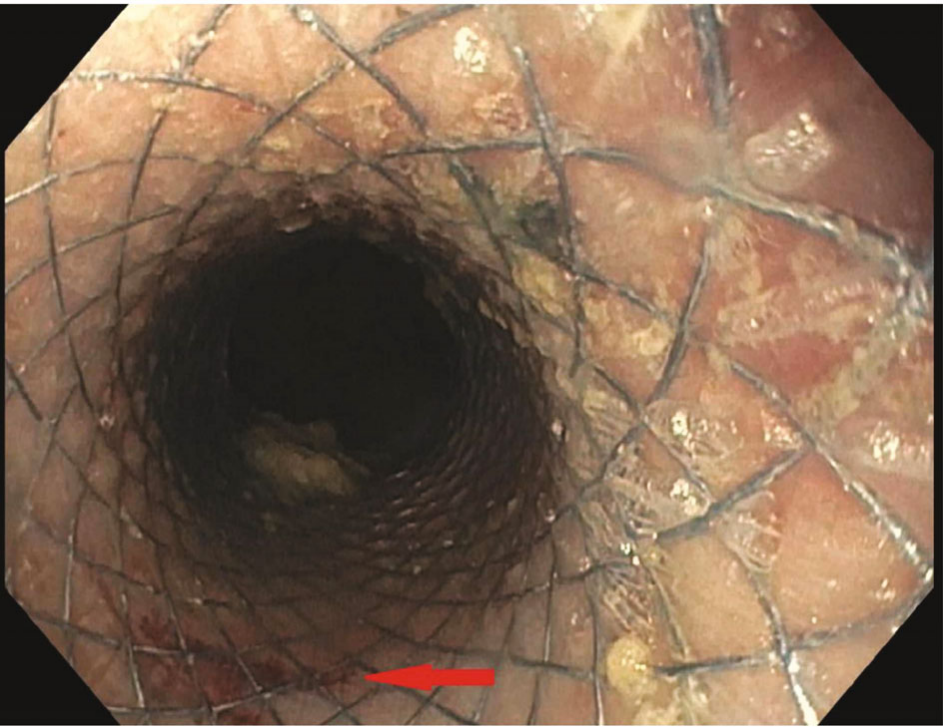
Endoscopic repair with a stent. The red arrow indicates the perforation.

An endoscopic approach for management of esophageal rupture was chosen as the patient was deemed too high risk for surgical intervention. After a month, the misleading ECG abnormalities resolved with sepsis management combined with esophageal stenting (Figures 3 and 4). Repeat endoscopy for surveillance showed evidence of healing with mild granulation tissue and mild inflammation. The previously placed stent was removed without any complications.

Two years later, the patient continues to follow up in the clinic and is doing well. He remains in sinus rhythm with resolution of his previously noted ST segment abnormalities.

## 3. Discussion

Boerhaave's Syndrome is a rare and deadly disease that has been diagnostically challenging since its original description in 1724. Symptoms are nonspecific and can mimic a number of other conditions including myocardial infarction, aortic dissection, pancreatitis, and pneumothorax among others. Furthermore, esophageal rupture is comparatively rare with an estimated incidence of only 3.1/1,000,000 per year [4]. These factors can lead to delays in diagnosis which is of great clinical significance as initiation of therapy in less than 24 hours from rupture is one of the greatest prognostic factors with respect to mortality [5].

Iatrogenic esophageal perforation was part of our differential because of the decision to pursue TEE. The patient had a history of chronic GERD that was untreated. Chronic untreated GERD can lead to mucosal irritation, injury, and even strictures in the esophagus. Probe manipulation during TEE of friable tissue can lead to esophageal perforation and bleeding [6]. Even though in population studies the majority of cases of esophageal perforation are iatrogenic, we feel it was unlikely in this case for multiple reasons [4]. First, the area of esophageal perforation was distal to the area of manipulation of the ultrasound probe. Additionally, the patient's clinical condition had already begun to deteriorate prior to the TEE being performed. These observations combined with the patient's history of recent vomiting left us confident this was truly spontaneous esophageal perforation. In retrospect, esophageal manipulation was risky. Had the TEE probe been advanced to the distal esophagus or transgastric and manipulated, we could have potentially propagated the esophageal perforation. This would have an increased risk of bleeding and seeding of the mediastinum with gastrointestinal flora resulting in sepsis. Sepsis is a major complication contributing to high morbidity and mortality [7]. Our patient had a renal transplant and was on immunosuppression, so collectively with infectious disease, the decision was made to obtain blood, urine, and sputum cultures before empirically starting Vancomycin, Cefepime, and Micafungin to cover for gastrointestinal flora which includes gram positives, gram negatives, anaerobes, and yeast.

CT angiography and transesophageal echocardiography are both excellent diagnostic studies to assess for aortic dissection but also carry their own risk. The patient's history of a renal transplant and the risk of contrast exposure led to the choice of performing a TEE. This case highlights the importance of considering esophageal pathology as a cause of chest pain in critically ill patients.

In this case, diagnosis was complicated by the presence of localized and evolving ST segment elevations in the setting of known ischemic heart disease. A review of literature returned a small number of case reports of esophageal perforation causing ST segment elevations on surface ECG [1, 8, 9, 10]. In all cases, cardiac biomarkers remained negative throughout. The ST segment changes seen appear to be caused by inflammation within the mediastinum as similar phenomenon has been described in descending necrotizing mediastinitis as well [11]. Despite these similarities, there are other details that make the current case unique. First and foremost, none of the other described cases in the literature involved a patient with known coronary artery disease. This factor weighed heavily in the decision-making process and ultimately led to a coronary angiogram despite negative cardiac biomarkers. The other unique factor in this case was the development of a junctional rhythm. This has not previously been described in the setting of esophageal perforation, and its pathophysiology remains unexplained. It remains unclear if the junctional rhythm was caused by the local inflammation itself or was a consequence of the systemic inflammatory response in an individual with severe cardiac disease.

In conclusion, we describe a case of Boerhaave's Syndrome that was associated with chest pain and localized ST segment elevation and the development of a junctional rhythm. Although rarer than other causes of chest pain, esophageal perforation should be considered in situations where there are ST segment changes with negative cardiac biomarkers as prompt recognition has significant impact on mortality. The association of a junctional rhythm with mediastinitis has never been described previously and requires further investigation to determine the etiology and clinical significance.
